# Improved Optoelectronic Properties and Temporal Stability of AZO/Cu/AZO Films by Inserting an Ultrathin Al Layer

**DOI:** 10.3390/nano15231780

**Published:** 2025-11-26

**Authors:** Haijuan Mei, Rui Wang, Jianming Deng, Yi Yu, Yimeng Song, Zhenting Zhao, Junfeng Zhao, Qiuguo Li, Zhaohui Guo, Cihong Lin, Weiping Gong

**Affiliations:** 1 Guangdong Provincial Key Laboratory of Electronic Functional Materials and Devices, Huizhou University, Huizhou 516007, China; haijuanmei@hzu.edu.cn (H.M.); xqf20081213@126.com (Y.Y.); ymsong@hzu.edu.cn (Y.S.); zhzhting@hzu.edu.cn (Z.Z.); 416zhaojunfeng@163.com (J.Z.); liqiuguo10@163.com (Q.L.); zhguo@hzu.edu.cn (Z.G.); 2 Guangxi Key Laboratory of Special Engineering Equipment and Control, Guilin University of Aerospace Technology, Guilin 541004, China; wanrui@guat.edu.cn; 3 Department of Materials Science and Engineering, Southern University of Science and Technology, Shenzhen 518000, China; 18927306664@163.com

**Keywords:** AZO/Al/Cu/AZO, microstructure, optoelectronic properties, temporal stability

## Abstract

An ultrathin Al layer was introduced into AZO/Cu/AZO films to further enhance the optoelectronic performance. The AZO/Al/Cu/AZO films were deposited on glass substrates by DC and RF magnetron sputtering; the microstructure and optoelectronic properties were analyzed by XRD, SEM, AFM, TEM, visible spectrophotometer, and Hall effect measurement system. The results indicated that the Al layer played a crucial role in modulating the crystallization behavior and optoelectronic properties of the films, exhibiting a distinct thickness-threshold effect. At an Al layer thickness of 1 nm, the film exhibited optimal optoelectronic performance, achieving a high *F_OM_* of 0.71 Ω^−1^, a high transmittance of 85%, and a low resistivity of 5.7 × 10^−5^ Ω·cm. However, when the Al layer thickness exceeded 1 nm, the crystallinity of the films deteriorated significantly, the grain boundary scattering and light absorption effect enhanced, leading to the deterioration of photoelectric properties. The introduction of the Al layer significantly improved the stability of the films, and the AZO/Al(2 nm)/Cu/AZO film exhibited the best temporal stability after being exposed to air for 20 months.

## 1. Introduction

Due to its good optoelectronic performance, transparent conductive oxides (TCOs) not only serve as a core transparent electrode material in the commercial optoelectronic devices such as solar cells and flat panel displays [1,2,3,4], but also show great potential in the field of energy-efficient windows [5]. With its outstanding optoelectronic properties, as well as high hardness and chemical corrosion resistance, indium tin oxide (ITO) films have been successfully used in the field of optoelectronic devices [6,7,8]. However, ITO films have disadvantages of high cost, high brittleness, poor bending resistance, and poor stability, limiting their further application in flexible optoelectronic devices. In comparison with ITO, aluminum-doped zinc oxide (AZO) has the advantages of being abundant, cheap, and non-toxic constituents, showing an enhanced optical transparency and thermal stability [9,10,11,12]. However, the resistivity remains relatively high for some special applications [13,14].

Recently, a multilayer structure of TCO/metal/TCO (TMT) has been proposed to improve the comprehensive optoelectronic performance. In a multilayer structure, the metal layer mainly provides a high electrical conductivity [15], while the TCO layer has a significant inhibitory effect on the light reflection from the metal layer, and the selective transparent effect can be achieved through the interference elimination of the incident visible light [16]. Moreover, the mechanical stability of multilayer films can be significantly enhanced by the introduction of a soft metal layer [17]. Thus, due to the perfect combination of high optical transparency and electrical conductivity, the TMT films have been widely investigated, such as AZO/Au/AZO [18], AZO/Ag/AZO [19], AZO/Cu/AZO [20,21], AZO/Ni/AZO [22], AZO/Mo/AZO [23]. Among these metals, Cu has been found to be the best alternative due to its high electrical conductivity and being cheaper than the noble metals Au and Ag. However, metal Ag or Cu atoms are prone to diffusion and oxidation at high temperatures, resulting in a sharp decrease in the optoelectronic properties at high temperatures [24]. To improve the high-temperature stability of TMT multilayer films, incomplete oxidation interface layers such as Al, Ti, and Ni are usually introduced to prevent the diffusion and oxidation of Ag or Cu atoms at high temperatures, while also playing a buffering and connecting role. For example, the insertion of a Ni interface layer effectively prevented the diffusion and oxidation of Ag atoms in the AZO/Ni/Ag/AZO multilayer films, then the optoelectronic stability was enhanced under a thermal environment [25]. The AZO/Ti/Cu/AZO multilayer films were deposited by magnetron sputtering, and found that the insertion of a Ti interface layer improved the crystallization of top top-layer AZO film, contributing to an increase in the optoelectronic and infrared properties [26]. In addition, the Ti layer also avoided the oxidation of metal Cu and improved the temporal stability. A similar result was also found for the AZO/Ti/Ag/AZO multilayer films; both the near-infrared transmittance and electrical conductivity were improved by the insertion of a Ti interface layer [27]. Moreover, the insertion of an Al interface layer was also found to prevent Ag atoms from diffusing into the AZO layer at high temperatures, thereby improving the thermal stability of the AZO/Al/Ag/Al/AZO multilayer films [28].

Thus, in this study, an ultrathin Al layer was introduced into the AZO/Cu/AZO multilayer films to serve as both an interface-optimizing layer and a diffusion barrier, thereby synergistically enhancing the optoelectronic properties and stability of the films. By systematically varying the thickness of the Al layer, this research aimed to elucidate its influence on the microstructure, optoelectronic properties, and temporal stability of the multilayers, thus providing an experimental basis and theoretical guidance for the design of TCO films with both high performance and high stability.

## 2. Experimental Details

### 2.1. Coating Deposition

**Figure 1:**
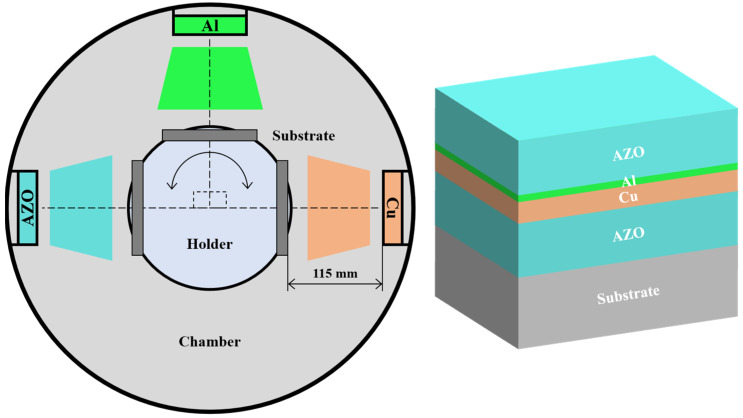
Schematic diagram of the deposition system and multilayer structure.

AZO/Al/Cu/AZO multilayer films were deposited on glass substrates by DC and RF magnetron sputtering using a ceramic AZO target (99.99% purity, ZnO:Al_2_O_3_ = 98:2 wt%, Ø76.2 × 4 mm^2^), metal Al and Cu targets (99.99% purity, Ø76.2 × 4 mm^2^). The multilayer films were fabricated by using an alternating magnetron sputtering system, as schematically illustrated in Figure 1. The deposition chamber was equipped with a rotational substrate holder positioned 115 mm from the sputtering targets. Prior to deposition, all substrates underwent a sequential ultrasonic cleaning process in acetone and ethanol, each for a duration of 20 min, followed by desiccation and subsequent mounting onto the holder. The chamber was initially evacuated to a base pressure of 8.0 × 10^−4^ Pa, and then the substrate temperature was heated to 200 °C. To ensure a clean surface for adhesion, an in situ Ar^+^ ion pre-sputtering etching was performed for 15 min at a bias voltage of 800 V. The sputtering process was conducted under a high-purity Ar flow of 150 sccm and a working pressure of 0.5 Pa. Both the bottom and top AZO layers were deposited by RF magnetron sputtering using a ceramic AZO target for 14 min, and the target power was 70 W. The Cu layer was deposited over the bottom AZO layer by DC magnetron sputtering using a metal Cu target for 52 s, and the target power was set at 30 W. Then, a thin Al layer was deposited over the Cu layer by RF magnetron sputtering using a metal Al target at a target power of 300 W, and the thickness of the Al layer was varied by using different deposition times of 0, 5, 10, and 15 s. The deposition parameters are listed in Table 1.

### 2.2. Coating Characterization

Surface and cross-sectional morphologies were examined using a scanning electron microscope (SEM, Tescan Vega 3 Xmu, Brno, Czech Republic), from which the thickness of the films was directly determined. Crystalline phase identification was performed by X-ray diffraction (XRD, Bruker D8 Advance, Karlsruhe, Germany) with Cu *K_α_* radiation in a θ–2θ mode. The resulting diffraction patterns were then analyzed to calculate the residual stress (via a biaxial strain model [29]), lattice parameter (via Bragg’s law [21]), and grain size (via the Scherrer equation [30]). Surface topography and roughness were quantified by atomic force microscopy (AFM, MFP-3D, Oxford, MS, USA) in contact mode over a 60 × 60 μm^2^ scan area. The nano-multilayer architecture was further investigated with a transmission electron microscope (TEM, Talos F200X, Thermo, Waltham, MA, USA), and the cross-sectional specimen was prepared via a dual-beam focused ion beam (FIB) system. Optical transmittance spectra were recorded in the 300–1000 nm wavelength range by using a visible spectrophotometer (723PCSR, Ruifeng, Guangzhou, China). The electrical properties were measured at room temperature using a Hall effect measurement system (CH-100, Cuihai, Beijing, China) based on the van der Pauw method.

## 3. Results

### 3.1. Microstructure

**Figure 2:**
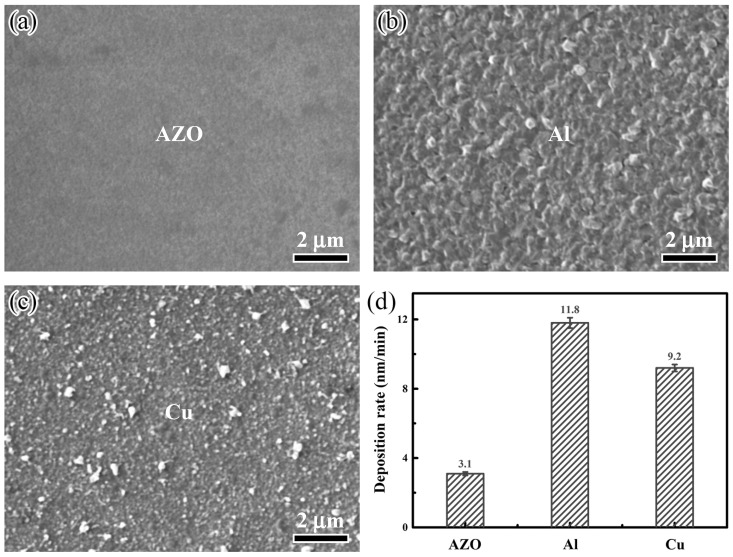
Surface SEM images and deposition rate of the monolayer films: (**a**) AZO film, (**b**) Al film, (**c**) Cu film, (**d**) deposition rate.

Figure 2 shows the surface SEM images and deposition rate of the monolayer films. In Figure 2a–c, it can be seen that the AZO film exhibited a relatively smooth surface, while the Al and Cu films showed obvious rough characteristics and distinct particle sense. This morphological variation primarily stems from the differences in material properties and deposition parameters. During the deposition of magnetron sputtering, the sputtering of the target material, the transport of particles, and the deposition and growth on the substrate jointly shape the final morphology of the film. As an oxide semiconductor, the growth process of AZO film tends to form regularly arranged grains. Combined with possible doping effects and optimization of deposition parameters, it contributes to its smooth surface, which is crucial for its application as a transparent conductive film, helping to reduce surface resistance and maintain high transmittance. In contrast, as pure metals, Al and Cu atoms typically exhibit higher surface energy and stronger nucleation driving forces. This enables their deposition process to more readily follow an island-growth pattern, forming isolated particles of varying sizes that create a rough surface. This rough and well-particle surface may introduce more scattering centers, thus affecting its optical properties. Under the conditions of 70 W, 300 W, and 30 W target power, the deposition rates of AZO, Al, and Cu films were 3.1 nm/min, 11.8 nm/min, and 9.2 nm/min, respectively, as shown in Figure 2d. Compared to metal Al and Cu films, the AZO film exhibited the lowest deposition rate. First, AZO is an oxide semiconductor; its sputtering rate is much lower than that of a metallic target. Second, the growth mechanism of oxide films tends to form a regular lattice structure, requiring more precise atomic alignment, which also limits the high deposition rate. Among these, the Al film has the highest deposition rate, which is directly related to the highest target power used. The high power significantly enhances the sputtering yield and produces a large number of high-energy particles, greatly improving the deposition rate. Thus, the thickness of nanolayer films can be precisely controlled by optimizing the deposition parameters and precisely controlling the deposition time, as listed in Table 2. When the thickness of the Al layer increased from 0 to 3 nm, the total thickness of AZO/Al/Cu/AZO multilayer films gradually increased from 94 to 97 nm, while the thickness of AZO and Cu films was set at 43 and 8 nm, respectively.

**Figure 3:**
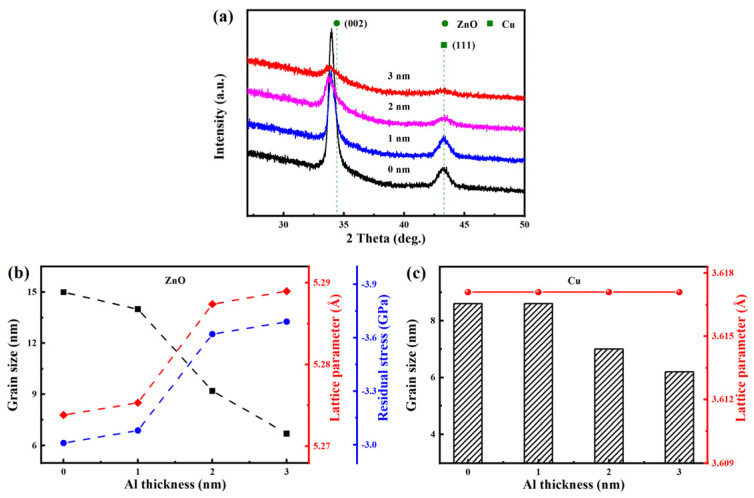
(**a**) XRD pattern of the AZO/Al/Cu/AZO films at various Al thicknesses, (**b**) grain size, lattice parameter, and residual stress of the AZO films, (**c**) grain size and lattice parameter of the Cu films.

Figure 3 displays the XRD pattern, grain size, lattice parameter, and residual stress of AZO/Al/Cu/AZO films. In Figure 3a, a strong ZnO (002) crystal plane diffraction peak was observed near 34.0°. This indicates that in the AZO/Al/Cu/AZO multilayer films, the AZO film presented a hexagonal wurtzite structure, and grew preferentially along the c-axis direction. Compared to the standard diffraction peak of ZnO (JCPDS 36-1451), the (002) diffraction peak shifted towards lower angles, primarily due to the presence of residual stress in the films, leading to an increase in the lattice parameter [31]. In Figure 3b, as the thickness of the Al layer increased from 0 to 3 nm, the residual stress and lattice parameter gradually increased from −3.01 GPa and 5.274 Å to −3.69 GPa and 5.289 Å, respectively. With the increase in Al thickness, the intensity of the ZnO diffraction peak gradually decreased, and then the grain size first slightly decreased from 15.0 to 14.0 nm, and then sharply reduced to 6.7 nm. It clearly indicated that the introduction of an ultrathin Al layer and its increased thickness significantly suppressed the crystalline quality of the top-layer AZO. When the Al layer was thin, its inhibitory effect was relatively limited. However, when the Al layer thickness exceeded 2 nm, the inhibitory effect became significant, resulting in a sharp decrease in grain size. This is significantly different from the AZO/Ti/Cu/AZO multilayer films reported in the literature [26]. With the increase in Ti layer thickness (2~18 nm), the grain size of AZO showed a trend of increasing and then decreasing. When the Ti layer was thin, the crystal growth time was short, and the crystallinity was poor. Therefore, it could not provide a good deposition environment for the top AZO layer, which affected its crystal growth, leading to a small grain size and poor crystallinity. In this study, the Al layer thickness was very thin (0~3 nm); its inhibitory effect on the crystallization of the top AZO layer was primarily attributed to nucleation effects, interfacial chemistry, and intrinsic stress induced by ion bombardment during sputtering.

In addition, a small diffraction peak appeared at approximately 43.3°, which can be attributed to the Cu (111) crystal plane, and the lattice parameter remained at 3.6171 Å, as shown in Figure 3c. Similarly, when the Al thickness increased, the intensity of the Cu diffraction peak gradually decreased, and the influence of Al layer thickness on the crystallization quality of the bottom layer Cu also showed an obvious threshold effect. When the Al layer was thin at 1 nm, it had no significant effect on the growth of the Cu, and the grain size remained unchanged at 8.6 nm. When the Al layer thickness increased to 2 nm and above, the intensity of the Cu diffraction peak was significantly weakened, and the grain size sharply decreased to 6.2 nm, indicating that the thicker Al layer also had a significant inhibitory effect on the crystallization quality of the underlying Cu layer. These may be explained by the following aspects. First, the thicker Al layer may have a physical cover or stress effect on the lower Cu layer during deposition. Although the Al layer is located on top of the Cu layer, its deposition may introduce vertical stress, which is transmitted through the Cu/Al interface and hinders the further growth of Cu grain, reducing the intensity of the Cu diffraction peak and refining the grain. Second, if interfacial diffusion occurs, the Al layer may also change the elemental composition or chemical environment at the top layer of Cu, which could also affect the crystalline state of Cu. Thus, although the Al layer was deposited later, its thickness still had a certain negative effect on the final crystallization quality of the underlying Cu through stress transfer and interface diffusion. Because the Al layer was very thin (0~3 nm), it was not sufficient to form a continuous and well-crystallized film, and no obvious Al diffraction peak was observed in the XRD pattern.

**Figure 4:**
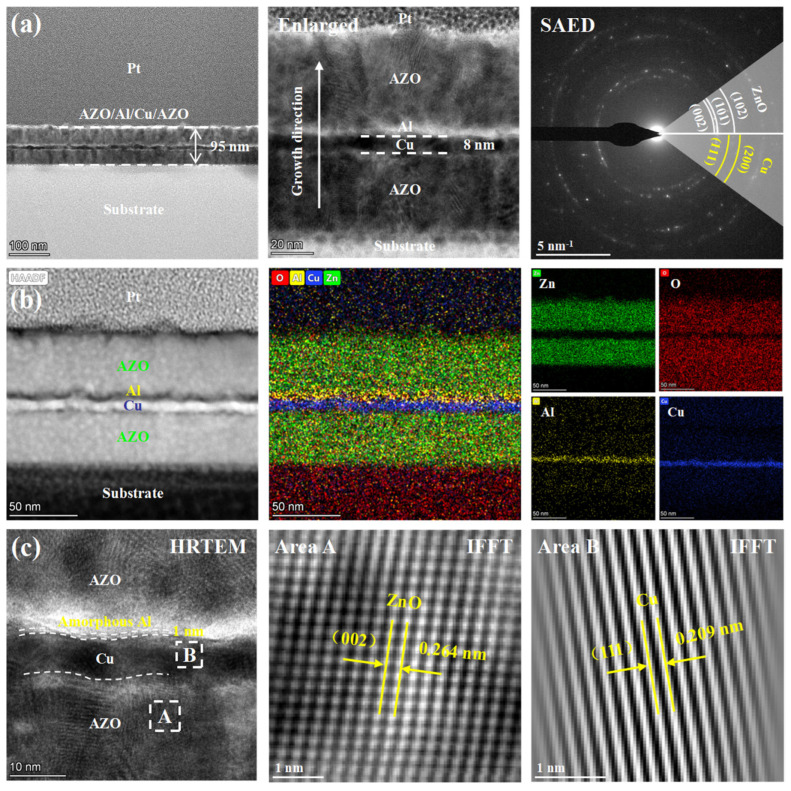
Cross-section TEM images of AZO/Al(1 nm)/Cu/AZO film: (**a**) Bright-field images, SAED pattern, (**b**) HAADF image and STEM mapping, (**c**) HRTEM image and IFFT pattern.

The microstructure and nano-multilayer structure of AZO/Al/Cu/AZO film were analyzed by TEM. Figure 4 displays the cross-section TEM images of AZO/Al/Cu/AZO film with an Al thickness of 1 nm. In Figure 4a, the overall thickness of the multilayer film is uniform and consistent, with a total thickness of about 95 nm. A clear and flat interface was formed between the film and the substrate, and the interface was dense. No structural defects such as holes, voids, or delamination were observed, indicating that the film was well-bonded to the substrate. From the high magnification bright-field image, the film presents a typical multilayer structure along the growth direction, in which the Cu layer thickness is 8 nm, and the continuity of the film is good. The selected area electron diffraction (SAED) pattern showed some diffraction rings, corresponding to the ZnO and Cu phases, which were in congruence with the XRD results. Because the Al layer is very thin, only 1 nm, no Al-related phase was observed in the SAED pattern. Such a thin thickness makes it difficult for Al atoms to arrange into a lattice structure with long-range order, resulting in extremely poor crystallinity and more likely to exist in the form of amorphous or small nanocrystals. In Figure 4b, the high-annular dark-field (HAADF) and scanning transmission electron microscope (STEM) mapping show the multilayer structure with a clear interlayer interface. The elements in each layer were distributed uniformly, and the elemental distribution of thin layers of Al and Cu can be clearly seen. This indicates that each layer grew in an orderly manner during deposition, and the interface was well controlled without obvious element mixing and diffusion. As shown in Figure 4c, some morphological undulations occurred at the upper and lower interfaces of the Cu layer, showing a wavy contour, which is directly attributed to the large surface roughness of the Cu film (in Figure 2), and the interface unevenness is more obvious at the nanoscale. Due to the difference in atomic number contrast, the Cu with a higher atomic number appears as a dark area in the bright-field image, while the Al appears as a bright area. The inverse fast Fourier transform (IFFT) analysis identified the existence of ZnO (002) and Cu (111) crystal planes, and measured the corresponding crystal plane spacing as 0.264 and 0.209 nm, respectively, which were consistent with the standard crystal plane spacing of the hexagonal wurtzite structure ZnO and face-centered cubic structure Cu.

### 3.2. Optoelectronic Properties

**Figure 5:**
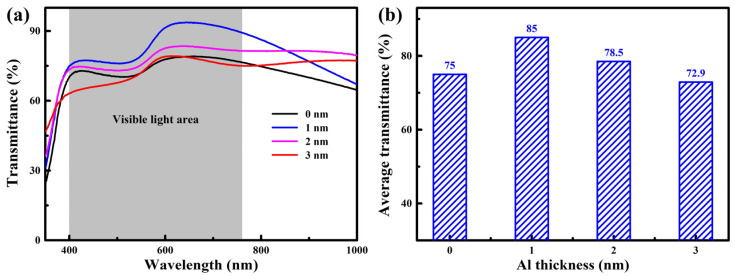
(**a**) Transmittance spectra, (**b**) average transmittance of the AZO/Al/Cu/AZO films.

Figure 5 shows the transmittance spectra and average visible light transmittance of the AZO/Al/Cu/AZO multilayer films at different wavelengths. As shown in Figure 5a, within the visible wavelength range of 400–760 nm, the transmission spectrum shows two characteristic peaks, indicating higher transmittance obtained in these two regions. This phenomenon is primarily attributed to the multiple interference effects within the film and the optical synergistic effect at the interfaces. With the increase in Al layer thickness, the transmission spectrum of the film showed a trend of first rising and then decreasing. In Figure 5b, without the introduction of an Al layer, the average visible light transmittance of the AZO/Cu/AZO multilayer film was 75.0%. When the Al thickness was optimized to 1 nm, the transmittance of AZO/Al/Cu/AZO film reached a peak of 85.0%, which can be attributed to the synergistic action of two mechanisms. On the one hand, the interference cancellation between the Al layer of appropriate thickness and the high-refractive-index AZO layer can effectively suppress reflection from the metal layer, thereby enhancing the overall transmittance. On the other hand, at an Al thickness of 1 nm, the multilayer film also exhibited good crystalline quality, with low internal defects and grain boundary density, which significantly reduced photon scattering loss. Similar phenomena have also been reported in the AZO/Ti/Cu/AZO films [26]. When the Ti layer thickness was 6 nm, the maximum transmittance of the film reached 87.9% due to the interference cancellation and good crystallinity. However, when the Al layer thickness was further increased, the transmittance of the multilayer films gradually decreased to 72.9%, which can be explained in two aspects. First, as indicated by the XRD results, when the Al layer thickness increased to 2 nm and above, the crystallinity of the films deteriorated sharply, and the grain size decreased significantly. This led to an increase in defects and grain boundaries, which enhanced photon scattering and consequently resulted in a decrease in transmittance [31]. Second, an excessively thick Al layer also enhanced the absorption of incident photons, thereby reducing the transmittance [21].

**Figure 6:**
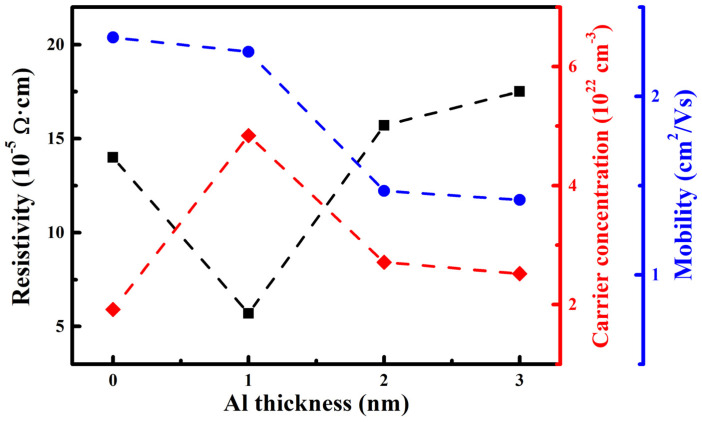
Resistivity, carrier concentration, and mobility of the AZO/Al/Cu/AZO films.

Figure 6 displays the electrical resistivity, carrier concentration, and mobility of the AZO/Al/Cu/AZO films with different Al layer thicknesses. When the Al layer thickness increased, the electrical resistivity first decreased and then increased, while the carrier concentration showed an opposite trend, first increasing and then decreasing, and the mobility steadily decreased. This observation elucidates the intricate role of Al layer thickness in governing the electrical conductivity of the thin film, a process governed by the interplay between carrier concentration and mobility. Specifically, when the Al layer thickness was increased from 0 to 1 nm, a marked reduction in resistivity, from 1.4 × 10^−4^ Ω·cm to 5.7 × 10^−5^ Ω·cm, was observed. This enhancement in the conductivity was mainly ascribed to a significant boost in carrier concentration. As a low work function metal, Al can provide additional free electrons at the interface, significantly increasing the carrier concentration. However, due to the poor continuity of ultrathin Al film, it is easy to form isolated island structures. These islands will form barriers in the carrier transport path and enhance the carrier scattering, resulting in a decrease in the mobility. Although the mobility was reduced, the increase in carrier concentration was greater than the negative impact of the decrease in mobility. The combined effect of the two factors significantly improved the overall conductivity of the film [25]. When the Al layer thickness further increased, the electrical resistivity of the films gradually increased to 1.8 × 10^−4^ Ω·cm at 3 nm, which was mainly caused by the simultaneous decrease in carrier concentration and mobility. Based on the above XRD results, with the increase in Al thickness, the crystallinity of AZO and Cu films decreased significantly, the grain size decreased, and the grain boundary density increased correspondingly. As the primary scattering center for carrier transport, grain boundaries significantly enhance carrier scattering probability through their barrier effect, leading to a decline in the carrier mobility [21]. Moreover, increased grain boundary density may introduce additional defect states that trap free carriers, further decreasing carrier concentration. Thus, the combined reduction in both carrier concentration and mobility leads to an increase in electrical resistivity of the films.

**Figure 7:**
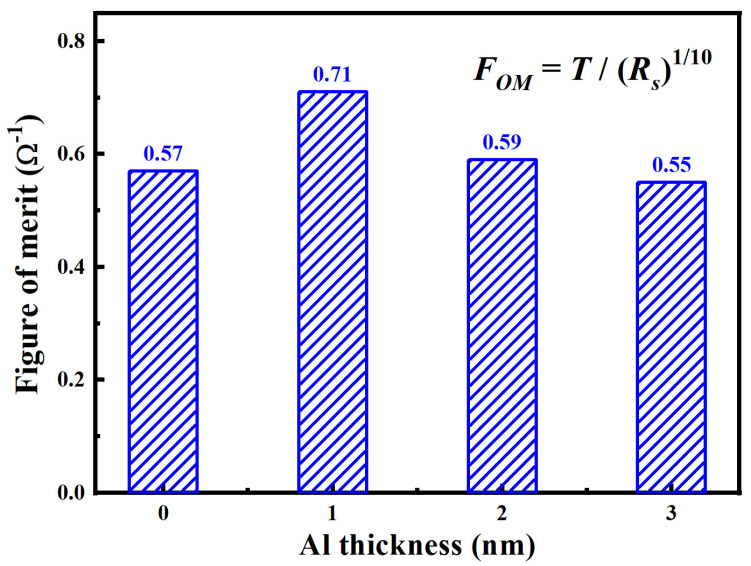
Figure of merit of the AZO/Al/Cu/AZO films at various Al thicknesses.

For transparent conductive film, the high-resolution figure of merit (*F_OM_*) serves as a key metric for evaluating its comprehensive optoelectronic performance. It is defined as the ratio of light transmittance to sheet resistance, which can reflect the conductivity and transmittance of the film [32,33]. Figure 7 displays the *F_OM_* of AZO/Al/Cu/AZO films with different Al layer thicknesses. With the increase in Al layer thickness, the *F_OM_* first increased and then decreased. When the Al layer thickness increased from 0 to 1 nm, *F_OM_* increased sharply from 0.57 to 0.71 Ω^−1^, exhibiting the best optoelectronic performance. This phenomenon was mainly attributed to the introduction of an ultrathin Al layer, which significantly enhanced the carrier concentration, thereby effectively reducing electrical resistivity. In addition, due to the interference cancellation effect, the transmittance of the film in the visible light region was optimized. However, when the thickness of the Al layer further increased, grain boundary scattering and light absorption effects became progressively stronger, leading to a decrease in the *F_OM_*. To evaluate the optoelectronic performance of AZO/Al/Cu/AZO films, Table 3 compares key parameters (transmittance, sheet resistance, and *F_OM_*) with those of other TMT films reported in the literature. First, compared to the AZO/Cu/AZO films in Ref. [21], the AZO/Cu/AZO film prepared in this work achieved a lower sheet resistance and a higher transmittance. This improvement was attributed to the enhanced crystallinity. After introducing a 1 nm thick ultrathin Al layer, the optoelectronic performance of AZO/Al/Cu/AZO film reached its best, and the *F_OM_* was higher than that of the AZO/Ni/Ag/AZO [25] and AZO/Ti/Ag/AZO [27] films. As compared to the AZO/Ti/Cu/AZO [26,34] films, the AZO/Al/Cu/AZO film achieved a higher optical transmittance of 85% using just a 1 nm Al layer. It further confirmed that the AZO/Al/Cu/AZO films exhibited excellent optoelectronic properties, and are expected to be a new type of transparent conductive material.

**Figure 8:**
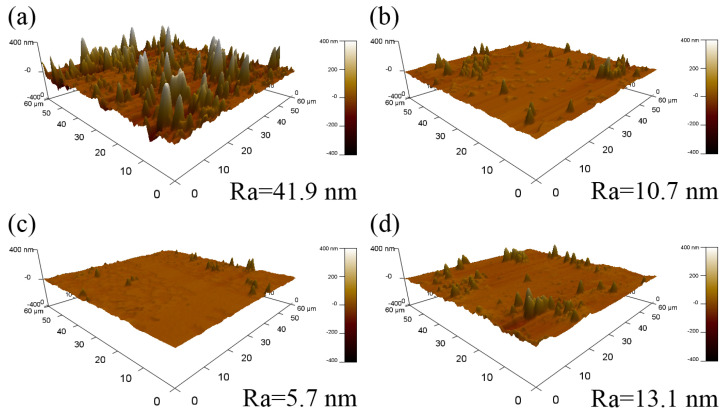
AFM images of the AZO/Al/Cu/AZO films after exposure to air for 20 months: (**a**) 0 nm, (**b**) 1 nm, (**c**) 2 nm, (**d**) 3 nm.

To systematically evaluate the long-term service stability of the films, this study conducted a 20-month atmospheric exposure experiment on the films, and the evolution of surface morphology was characterized using AFM. As shown in Figure 8, the surface of the AZO/Cu/AZO film without an Al interlayer underwent severe degradation, with its roughness increasing sharply to 41.9 nm. This drastic change can be attributed to the outward diffusion of Cu atoms through the grain boundaries or defects of the AZO layer, and then accumulated and oxidized on the surface, ultimately forming macroscopic particles. In contrast, the introduction of an Al layer played a decisive role in enhancing the temporal stability. The ultrathin Al layer acted as an efficient diffusion barrier and a chemical passivation layer. First, it physically suppressed the outward diffusion of Cu atoms. Second, the Al surface spontaneously formed a dense Al_2_O_3_ passivation film in air [35,36], which further isolated the internal diffusion of O and the external diffusion of Cu. With increasing Al layer thickness, the surface roughness of the films exhibited a non-monotonic trend, first decreasing and then increasing. When the Al layer thickness was 2 nm, its protective effect was optimal, and the surface roughness decreased to a minimum of 5.7 nm. This phenomenon was closely correlated with the evolution of the crystalline quality of the AZO and Cu layers. When the Al layer thickness increased, the grain sizes of AZO and Cu gradually decreased. The induced moderate grain refinement formed a dense grain boundary network and then effectively suppressed the outward diffusion of Cu atoms, thereby reducing surface roughness. However, when the Al layer thickness increased to 3 nm, the crystalline quality of AZO and Cu layers deteriorated excessively, generating an abundance of grain boundaries. These high-density grain boundaries, in turn, provided fast diffusion pathways for Cu atoms, which weakened the barrier effect and caused the surface roughness to rise again to 13.1 nm.

**Figure 9:**
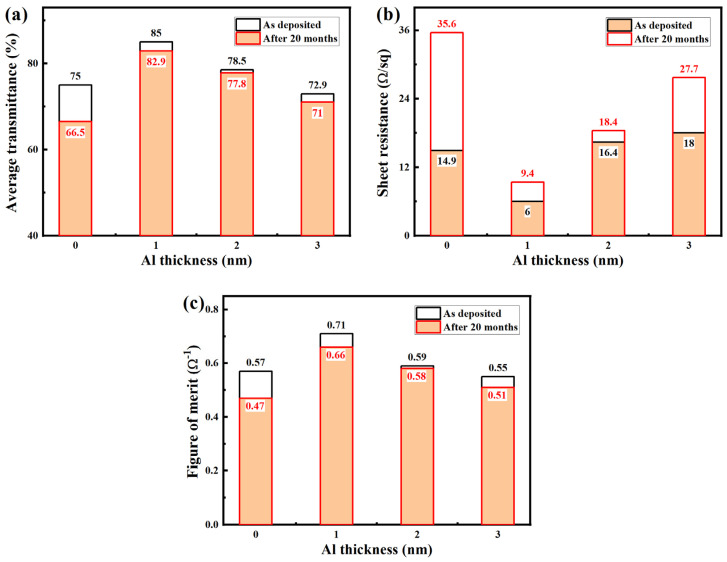
Average transmittance (**a**), sheet resistance (**b**), and figure of merit (**c**) of the AZO/Al/Cu/AZO films before and after exposure to air for 20 months.

Figure 9 illustrates the evolution of the optical transmittance, sheet resistance, and *F_OM_* for AZO/Al/Cu/AZO multilayer films before and after 20 months of exposure to ambient air. As shown in Figure 9a, the average transmittance of the AZO/Cu/AZO film decreased significantly from 75.0% to 66.5%. In contrast, the introduction of an Al layer effectively suppressed this degradation in transmittance; a similar phenomenon was also reported for the AZO/Ti/Cu/AZO films [26]. With increasing Al layer thickness, the attenuation of transmittance exhibited a non-monotonic trend, first decreasing and then increasing. When the Al layer thickness reached 2 nm, the film demonstrated optimal stability, with its transmittance slightly decreasing from 78.5% to 77.8%. This excellent performance was closely correlated with the evolution of the surface morphology of the films (Figure 8). The lower surface roughness effectively suppressed diffuse reflection and scattering of visible light, thereby reducing optical losses. In Figure 9b, the sheet resistance of the AZO/Cu/AZO film increased sharply from 14.9 Ω/sq to 35.6 Ω/sq after the 20-month exposure. Similarly, the incorporation of an Al layer significantly mitigated the deterioration of sheet resistance, and the extent of this increase also followed a non-monotonic trend. At an Al layer thickness of 2 nm, the sheet resistance showed only a minor increase from 16.4 Ω/sq to 18.4 Ω/sq, exhibiting the best stability. As previously discussed, in the multilayer structure, the ultrathin Al layer served a dual function as both a physical diffusion barrier and a chemical passivation. The dense Al_2_O_3_ passivation film formed therefrom effectively inhibited the outward diffusion of Cu atoms and blocked the ingress of external O [35], thus preventing the oxidation of the Cu layer and avoiding the consequent degradation of electrical conductivity [26]. As presented in Figure 9c, the *F_OM_* of the AZO/Cu/AZO film decreased sharply from 0.57 to 0.47 Ω^−1^ after long-term exposure. However, the temporal stability of the films was significantly improved by the introduction of an Al layer. When the Al layer thickness reached 2 nm, the *F_OM_* decreased slightly from 0.59 to 0.58 Ω^−1^, exhibiting the best temporal stability. The AZO/Al(1 nm)/Cu/AZO film still exhibited the best optoelectronic performance, maintaining the highest *F_OM_* of 0.66 Ω^−1^ after exposure to air for 20 months.

## 4. Conclusions

In this study, by inserting an ultrathin Al layer into the AZO/Cu/AZO film, both the optoelectronic properties and temporal stability of the films were significantly improved. The results revealed that the Al layer played a crucial role in modulating the crystallization behavior of the films, exhibiting a pronounced thickness threshold effect. When the thickness of the Al layer exceeded 1 nm, both the crystallinity and grain size of the AZO and Cu layers were significantly reduced. With the increase in Al layer thickness, the transmittance of the films first increased and then decreased, whereas the resistivity exhibited the opposite trend. Benefiting from the interlayer interference cancellation effect and an increased carrier concentration, the AZO/Al(1 nm)/Cu/AZO film exhibited the best overall optoelectronic performance, achieving an extremely high *F_OM_* of 0.71 Ω^−1^, a high transmittance of 85%, and a low resistivity of 5.7 × 10^−5^ Ω·cm. Regarding temporal stability, the introduction of the Al layer played a decisive role in enhancing the stability of the films. The ultrathin Al layer acted as an effective diffusion barrier and chemical passivation layer, which successfully suppressed the outward diffusion of Cu atoms. When the Al layer thickness was 2 nm, the film exhibited the optimal temporal stability after exposure to air for 20 months.

## Figures and Tables

**Table 1:** Deposition parameters of AZO/Al/Cu/AZO films.

Parameters			
Base pressure (Pa)	8.0 × 10^−4^		
Substrate temperature (°C)	200		
Working pressure (Pa)	0.5		
Target to substrate distance (mm)	115		
Target material	AZO	Al	Cu
Unbalanced magnetron sputtering	RF	RF	DC
Target power (W)	70	300	30
Deposition time	14 min	0, 5, 10, 15 s	52 s

**Table 2:** Monolayer thickness and total thickness of AZO/Al/Cu/AZO films.

Monolayer Thickness (nm)				Total Thickness (nm)
AZO	Al	Cu	AZO	
43	0	8	43	94
43	1	8	43	95
43	2	8	43	96
43	3	8	43	97

**Table 3:** Comparison of transmittance (*T*), sheet resistance (*R_s_*), and *F_OM_* with other literature.

Multilayer Films	Ag/Cu (nm)	Ni/Ti/Al (nm)	*T* (%)	*R_s_* (Ω/sq)	*F_OM_* (Ω^−1^)	Refs.
AZO/Cu/AZO	10	0	72.1	29.6	0.51	[21]
AZO/Ni/Ag/AZO	8	4	75.0	7.0	0.62	[25]
AZO/Ti/Ag/AZO	12	1	78.1	7.2	0.64	[27]
AZO/Ti/Cu/AZO	30	10	87.3	8.1	0.71	[26]
AZO/Ti/Cu/AZO	7	3	82.1	4.3	0.71	[34]
AZO/Cu/AZO	8	0	75.0	14.9	0.57	This work
AZO/Al/Cu/AZO	8	1	85.0	6.0	0.71	

## Data Availability

Data are contained within the article.
